# Bioinformatics in Africa: The Rise of Ghana?

**DOI:** 10.1371/journal.pcbi.1004308

**Published:** 2015-09-17

**Authors:** Thomas K. Karikari

**Affiliations:** Department of Science Laboratory Technology, School of Applied Science and Technology, Wa Polytechnic, Wa, Ghana; Pierre and Marie Curie University (UPMC), FRANCE

## Abstract

Until recently, bioinformatics, an important discipline in the biological sciences, was largely limited to countries with advanced scientific resources. Nonetheless, several developing countries have lately been making progress in bioinformatics training and applications. In Africa, leading countries in the discipline include South Africa, Nigeria, and Kenya. However, one country that is less known when it comes to bioinformatics is Ghana. Here, I provide a first description of the development of bioinformatics activities in Ghana and how these activities contribute to the overall development of the discipline in Africa. Over the past decade, scientists in Ghana have been involved in publications incorporating bioinformatics analyses, aimed at addressing research questions in biomedical science and agriculture. Scarce research funding and inadequate training opportunities are some of the challenges that need to be addressed for Ghanaian scientists to continue developing their expertise in bioinformatics.

## Introduction

The development of bioinformatics as a discipline has provided biological scientists with many important insights into the functioning and composition of biological systems [1–3]. Together with tools and methods developed within bioinformatics, these insights provide essential foundations for basic and applied research in current biomedical science, health care, and agriculture [3–8]. A notable example is the exponential growth recorded in the amount and diversity of genomic sequence data in recent years; significant events in this area include the sequencing of the *Haemophilus influenza* genome in 1995 [9] and the publishing of an initial draft of the human genome in 2001 [10], followed by the sequencing of a large number of other species and variants of the same species. For instance, 23,565 genome sequencing projects (permanent drafts) and 6,653 completed sequencing projects were listed in the Genomes OnLine Database as of June 2015, with 29,940 more projects ongoing at the time (https://gold.jgi-psf.org/index; accessed 10 June 2015). In all, sequencing data from 59,909 organisms were documented in the database. These numbers are expected to increase because of ongoing attempts to sequence genomes of different populations and organisms worldwide; an example is the 100,000 Genomes Project, which is aimed at sequencing 100,000 whole human genomes in England to better understand the link between rare variants and disease (http://www.genomicsengland.co.uk). Although these large volumes of information are valuable resources for the scientific community, the extremely rapid growth in database size also brings difficulties in analysing and deriving inferences from such data [1,4,11,12]. Computational research has become essential in the postgenomic era to help organise and store bioinformatics data, ensuring their retrieval and allowing further processing and analysis. This contributes towards improved understanding of the regulation and functioning of biological processes [3,4,11,12].

Over the last three decades, bioinformatics has grown into a scientific discipline [3,13]. A large proportion of the progress in this field has come from scientifically advanced countries, leaving many developing countries behind [1,2]. Recently, however, the application of bioinformatics has been improving in the developing world, with modest developments reported from several countries [1,2,13–18]. Here, I present an analysis of the incorporation of bioinformatics into biological science education and research in Ghana and how this is contributing to developing the discipline in Africa. I also discuss approaches that have been employed to strengthen capacity for bioinformatics, the challenges faced by scientists, and the opportunities for further development of the discipline.

## Bioinformatics in Africa

The challenges that Africa faces, including those of increasing disease burden, food insecurity, and malnutrition, can be addressed by improving the application of modern science, technology, and innovation (STI) to serve specific nations and the continent at large [19–21]. Many African countries are battling with serious challenges such as neglected tropical diseases (NTDs), human immunodeficiency virus/acquired immunodeficiency syndrome (HIV/AIDS), tuberculosis, and malaria [22–25]. STI approaches have been used to address these challenges, and the approaches have been shown in some cases to lead to improvements [26–31]. Considering these improvements, a potentially viable approach to enhance STI use would be to build a concerted Africa-wide agenda to constitute a framework to strengthen the continent’s capacities to develop, harness, and apply STI for developmental purposes [19,21].

One area of STI that has been employed to improve research in Africa is bioinformatics [13,32]. Scientists in Africa are well positioned to contribute to bioinformatics, partly because infrastructural requirements for its use are relatively less expensive compared to similar activities conducted in bench-research-intensive disciplines [1]. Bioinformatics training can also be relatively inexpensive; the main costs usually involve training and information technology infrastructure, making the discipline a good use of scarce funding opportunities [33]. In recent years, the cost of genome sequencing has been decreasing [4,12]; the scientific community is close to achieving a much anticipated target of US$1,000 in the $1,000 genomes project [34]. These cost reductions in high-throughput sequencing technologies suggest that more African laboratories can now acquire such resources to advance their research. Also, it is becoming easier to access the Internet in Africa; the average broadband speed and Internet penetration rates have been increasing in recent times [13]. Increasing both Internet speed and access makes bioinformatics tools and databases available to, and usable by, a wider range of researchers across the continent [13]. The improved Internet connectivity also suggests that the location of the bioinformatics experts (whether rural or urban) is of little consequence to their output, making the discipline a possible path to bring state-of-the-art scientific research to many parts of Africa.

Currently, the demand for scientists with bioinformatics expertise in Africa outweighs the supply [1,13,32,35]. The lack of scientists trained in bioinformatics means that increasing the amount of training could be a good way of addressing the shortfall [13,32]. For the continent to improve its benefits from bioinformatics, more scientists should be trained to be able to effectively carry out large-scale computational experiments and analyses [32,36]. Building bridges (between African scientists and between African and non-African scientists) is one of the approaches that can help to improve Africa’s participation in bioinformatics and facilitate the development of scientific capacity and productivity in this discipline [13,32,37,38].

Organisations such as the African Society of Human Genetics (AfSHG, http://www.afshg.org) and the African Society for Bioinformatics and Computational Biology have been contributing to building bioinformatics capacity in Africa, through efforts such as the provision of training programmes and the establishment of research facilities [13,32]. Recently, the AfSHG partnered with the United States National Institutes of Health and the United Kingdom-based Wellcome Trust to establish the Human Heredity and Health in Africa (H3Africa) project (http://www.h3africa.org) [13,32,36]. H3Africa is one of the biggest scientific capacity-building initiatives in Africa and has so far seen the disbursement of several millions of dollars in research grants to African scientists [32,36]. One of the highlights of the H3Africa project is the development of a pan-African bioinformatics network called H3ABioNet, which seeks to build capacity for bioinformatics applications on the continent [13]. It is also hoped that the ten-year Science, Technology and Innovation Strategy for Africa plan adopted by the African Union with the aim of prioritising the use of scientific research to drive the continent’s socioeconomic development will contribute to the further development of contemporary fields like bioinformatics [39].

Much of the progress made in bioinformatics in Africa has come from a few countries, including South Africa, Kenya, and Nigeria [1,13–15,18,40]. South Africa has established research institutes, funding, and training initiatives to promote bioinformatics [13,15,18]. Bioinformatics in Africa is said to have started from South Africa, with the establishment of the South African National Bioinformatics Institute and the now defunct National Bioinformatics Network [13,18]. Scientists in Kenya have also been making progress in developing intellectual capacity for bioinformatics, for example, through research and training activities conducted at institutions such as the Biosciences Eastern and Central Africa (http://hub.africabiosciences.org), the International Livestock Research Institute (ILRI), and the International Centre of Insect Physiology and Ecology (ICIPE). Researchers in Kenya and South Africa and their collaborators recently sequenced and assembled the genome of the tsetse fly—the vector of human African trypanosomiasis, a devastating NTD [41]. In Nigeria, bioinformatics research groups have been constituted, and bioinformatics techniques have been applied to address local research questions, mainly focusing on malaria [14]. During the recent outbreak of the Ebola virus disease (EVD) in West Africa, Nigerian scientists, together with their international partners, provided a description of the disease epidemiology through whole-genome sequence analysis of affected patients and unaffected controls [42]. In this report, it was suggested that the EVD outbreak might have resulted from a previous outbreak in Central Africa [42]. Such studies are expected to help provide insights into disease mechanisms and advance disease control, with other important implications for biological research across the continent [40–42]. Aside from these leading countries, Ghana is another country making progress in bioinformatics.

## Bioinformatics in Ghana

### State of bioinformatics in Ghana

Bioinformatics in Ghana is in the developmental stages. One of the first activities was in the year 2003 when the country hosted the inaugural conference of the AfSHG, which aimed “to provide opportunities for networking and collaboration among professionals working on genetic and genomic issues relevant to Africa” [43]. Since then, considerable improvements have been made.

### Short courses and workshops

There have been a number of short-term bioinformatics training programmes offered in Ghana. These programmes have been mainly targeted at beginners, helping to provide them with foundational skills and preparing them for advanced bioinformatics training. Institutions leading the organisation of such courses include the University of Ghana (UG, http://www.ug.edu.gh/), the Noguchi Memorial Institute for Medical Research (NMIMR, http://www.noguchimedres.org), and the Kumasi Centre for Collaborative Research in Tropical Medicine (KCCR, http://www.kccr-ghana.org/). The Department of Biochemistry, Cell and Molecular Biology (BCMB) at UG hosted the fifth American Society of Cell Biology’s annual West African regional workshop series on infectious diseases in summer 2013 [44]. Functional genomics and bioinformatics formed part of this training course [44]. Through a partnership with the University of Cambridge, UK, the BCMB organised a ten-day workshop on diagnostic development for NTDs in July 2014, during which aspects of bioinformatics were taught. There are two H3ABioNet nodes in Ghana: one at NMIMR and the other at KCCR [32]. The H3ABioNet node at NMIMR recently organised bioinformatics workshops in Accra. Topics treated included introductions to bioinformatics and biological databases, molecular genetics, biological text mining, and computing for biological scientists [45]. A workshop on “Sequence analysis: patterns, similarities and differences discovery from genomic data” was also held at the KCCR node in November 2014.

### University programmes

Presently, no Ghanaian university offers bioinformatics as a degree programme on its own. Attempts to introduce bioinformatics into the curricula of Ghanaian universities have focused on integrating aspects of the discipline into existing life science degree programmes. For instance, the University of Cape Coast (UCC, http://www.ucc.edu.gh) has bioinformatics modules as part of the Bachelor of Science programmes in Biochemistry and in Molecular Biology and Biotechnology [46,47]. The BCMB department at UG also provides training in “advanced topics in bioinformatics” to postgraduate students [48].

### International projects, collaborations, and networks

Ghanaian researchers have been involved in a number of international projects involving bioinformatics. One of these is the H3Africa initiative. As of June 2014, Ghanaian scientists and institutions were involved in five H3Africa-funded multicentre projects in health-related genomics research [32]. Specifically, these projects included three multicountry collaborative studies, a project to explore public perceptions and attitudes to genomics-based interventions for sickle cell disease, and another on developing the H3ABioNet pan-African bioinformatics network [32]. The three H3Africa-supported collaborative research efforts involving Ghana included the (1) University of the Witwatersrand-INDEPTH Network-H3Africa (Wits-INDEPTH-H3Africa) collaborative centre, which aimed to build a sustainable team of African scientists to further the understanding of the role of genetic, epigenetic, and environmental factors in obesity and its related disorders (2) H3Africa Kidney Disease Research Network, which was targeted at employing genomic technologies to improve kidney disease research in Africa, and (3) Stroke Investigative Research and Educational Network, which had the goal of furthering the existing understanding of genetic and epigenetic factors that contribute to stroke pathology in Africa [32,49]. To improve its capacity for the use of bioinformatics, the H3ABioNet node at NMIMR recently recruited a bioinformatician to support its research activities [50].

Aside from H3Africa, Ghana has been involved in the European Union (EU) Seventh Framework Programme (FP7) projects in Africa [51]. The country had 54 contributions to the EU-funded programme and was ranked as the sixth most successful African country in terms of participation [51]. FP7 examples in Ghana included the Enhanced Protective Immunity Against Filariasis (E PIAF) project, which sought to (1) apply transcriptomics and bioinformatics to identify molecular targets of protective immunity against filariasis and (2) use bioinformatics and microarray technologies to obtain further understanding into protective immunity in the disease [51]. The Kwame Nkrumah University of Science and Technology (KNUST, http://www.knust.edu.gh) was a partner in the E PIAF project. In addition, over a dozen Ghanaian scientists have benefitted from Marie Curie funding, also from the EU [52].

### Analysis of Ghanaian bioinformatics publications

To provide information on the recent bioinformatics research activities and capacity, the PubMed, Web of Science, and SCOPUS databases were searched to identify peer-reviewed bioinformatics-related articles authored by Ghanaian scientists and published within the period of 2004–2014. This literature search aimed to do the following:

1. Provide an indication of recent efforts of applying bioinformatics in Ghana
2. Identify research areas in which bioinformatics applications have been focused
3. Identify Ghanaian institutions leading research in the discipline

**Fig 1:**
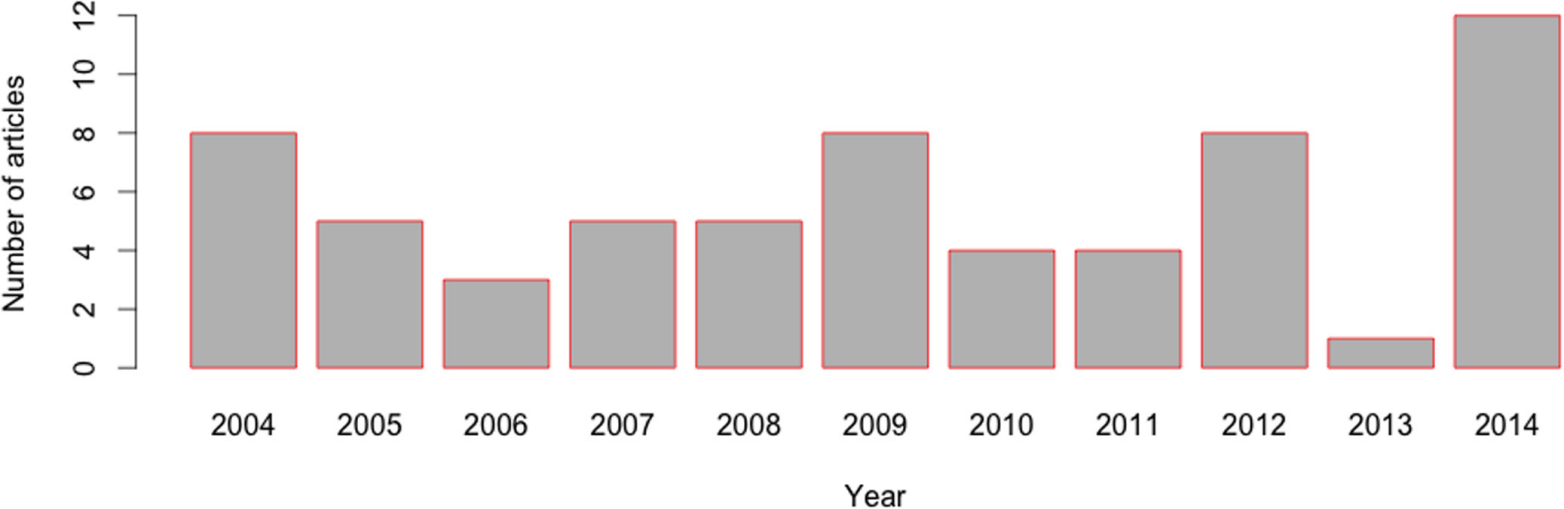
Bioinformatics-related publications authored by scientists affiliated with Ghanaian institutions. As a measure of bioinformatics research output in Ghana, the search terms “next-generation sequencing Ghana,” “computational biology Ghana,” “bioinformatics Ghana,” “genomic Ghana,” and “*in silico* Ghana” were used to obtain peer-reviewed research articles published between 2004 and 2014 and indexed in the PubMed, Web of Science, and SCOPUS databases (refer to the main text for article inclusion criteria).

The search terms “next-generation sequencing Ghana,” “computational biology Ghana,” “bioinformatics Ghana,” “genomic Ghana,” and “*in silico* Ghana” were used. Articles were sorted for duplication and also against the following inclusion criteria: the article was written in English, employed bioinformatics technique(s) (according to the definition of [53]), at least part of the study reported was conducted in Ghana or on samples/subjects from Ghana, and a minimum of one author was affiliated with a Ghanaian institution. In total, 63 articles were obtained (Fig 1, S2 Table). This outcome is indicative and may have missed other articles. The results showed that bioinformatics techniques have been applied in research in areas such as malaria, HIV/AIDS, hepatitis, tuberculosis, diabetes, cancer, NTDs, and crop research (Table 1, S1 and S2 Tables). Most of the articles retrieved emanated from collaborative projects between scientists in Ghanaian institutions and between those in institutions in Ghana and their counterparts abroad. Importantly, Ghanaian scientists took leading roles (first and/or corresponding author roles) in many of these studies. These include studies that investigated the regulatory roles of interleukins and immunoglobulins in malaria pathogenesis [54], the association of specific receptor allelic polymorphisms with clinical malaria [55], HIV-1 drug-resistant mutations in Ghanaian patients [56,57], human betacoronavirus-related viruses in bats [58], and the efficacy of an onchocerciasis treatment [59]. Others were studies into the genetic diversity of chicken populations in Ghana [60], the genomes of viruses that cause cassava mosaic disease [61], tuberculosis isolate genotyping [62], the genetic diversity in cowpea [63], the dynamics of maize streak virus genotypes [64], and the genetic characterisation of cocoyam accessions [65]. Table 1 summarises research areas in Ghana in which bioinformatics has been utilised, while S1 Table gives an extensive list of institutions leading bioinformatics-related research in the country, their research foci, institutional websites, and sample publications. Bioinformatics-related peer-reviewed publications authored by scientists from Ghana are provided in S2 Table.

**Table 1:** Major research areas in which bioinformatics has been applied in Ghana.

Research area	Research focus
Tuberculosis	Genotyping tuberculosis isolates; genetic susceptibility to, and protection from, tuberculosis; genomic diversity and evolution of *Mycobacterium ulcerans*.
Neglected tropical diseases	Genetic resistance to *Onchocerca volvulus* infections, polymorphisms associated with Buruli ulcer.
Malaria	Evolution of malaria-protective alleles in Africa, genetic basis of resistance to malaria drugs, genetic resistance to severe malaria, polymorphisms associated with malaria, genetic basis of insecticide resistance in mosquitoes.
Cancer	Genome-wide association studies of prostate cancer risk in West African men.
Visual sciences	Genome-wide scan for quantitative traits for intraocular pressure, optineurin coding variants in primary open-angle glaucoma.
Animal research	Genetic diversity of village chickens across Ghana, genomic sequencing of peste des petits ruminant virus, molecular epidemiology of *Neisseria meningitidis* infections.
HIV/AIDS and other viral diseases	Molecular epidemiology of HIV, betacoronaviruses-related viruses in bats, molecular characterisation of hepatitis B virus in Ghana, mother-to-child transmission of hepatitis B infections.
Diabetes, hypertension, and obesity	Genetic susceptibility to diabetes mellitus, polymorphisms linked to type 2 diabetes, genome-wide search for phenotypic traits linked to obesity, multilocus analysis of hypertension.
Inflammatory responses	Genetic susceptibility to proinflammatory responses.
Ahaptoglobinaemia	Genetic polymorphisms associated with ahaptoglobinaemia and hypoahaptoglobinaemia.
Renal function	Genome-wide studies of renal function phenotypes.
Crop research	Transmission of cacao pollen shoot viruses, genetic diversity of the shea tree, genetic diversity of cocoyam and cowpea, maize streak virus distribution across Ghana, genomic sequencing of viruses that cause cassava mosaic disease.

^1^ HIV/AIDS, Human Immunodeficiency Virus/Acquired Immunodeficiency Syndrome.

### Challenges and prospects of bioinformatics in Ghana

Bioinformatics challenges in the country, as well as suggested measures to improve the discipline, are provided below.

### Bioinformatics infrastructure is in short supply

Even though bioinformatics requires considerably less infrastructural investment than similar activities conducted in bench science research, even minor costs can be obstacles in many developing countries [1]. Effective bioinformatics applications require essential resources such as powerful computer systems, high-speed Internet connectivity, and continuous electrical power supply [1,33,66]. In Ghana, these resources are not usually available; electrical power failure, unreliable Internet connections, and lack of high-speed computer systems are some of the serious impediments to the effective development of the discipline.

Other challenges include the dearth of dedicated bioinformatics teaching and research facilities. Many Ghanaian research and higher education institutions (RHEIs) lack bioinformatics teaching and research infrastructure such as laboratories. Even within institutions in which bioinformatics is incorporated into degree programmes, dedicated laboratories for computational research are hard to find. The common practice is that bioinformatics teaching and research take place in shared computer laboratories. These shared facilities serve many students and staff members and are difficult to access because of the high demand for them [13]. Furthermore, the specifications and speed of the computers in such laboratories are often too low for bioinformatics applications [13]. Considering the fact that bioinformatics activities usually require powerful Internet connections and dedicated computers of high specification [13,66], the provision of well-resourced laboratories specifically for bioinformatics use would help to develop the discipline in Ghana.

One advantage of bioinformatics is the availability of several free, open-source resources (FOSR). FOSR allow for a virtual learning model of education to be applied to bioinformatics, allowing remote access to teaching materials [67]. Some of these FOSR directories are provided in S3 Table. Ghana can adopt such resources to improve bioinformatics knowledge. Small-scale hobbyist computers can also be adopted, including the credit card-sized single-board computer systems made by the Raspberry Pi Foundation and Beagleboards, with prices starting from about US$25 and US$55, respectively [68]. Although these computers may not be ideal for data-intensive applications in terms of processing power and storage space, they could be useful for several basic purposes, including teaching.

### Scientists with expertise in bioinformatics are lacking in Ghana

Whilst a few institutions have some scientists with appreciable knowledge in bioinformatics (S1 Table), most Ghanaian institutions lack this human resource capacity. This lack of expertise may adversely affect the quality of training. Moreover, an important capacity that is lacking in Ghana is the technical skills to analyse so-called “big data.” Expertise in data analysis is required to clean, transform, and model data, in order to make inferences, suggest conclusions, and inform research directions [6,69]. With the anticipated increase in genomic data from African populations, it is critical that attention is paid to world-class training in data analysis [13,32,49]. While some universities in Ghana are nurturing ambitions to start bioinformatics degree programmes, the short supply of expertise remains a major challenge. Pragmatic approaches should therefore be developed to train more people so they can lead teaching and research in bioinformatics.

**Fig 2:**
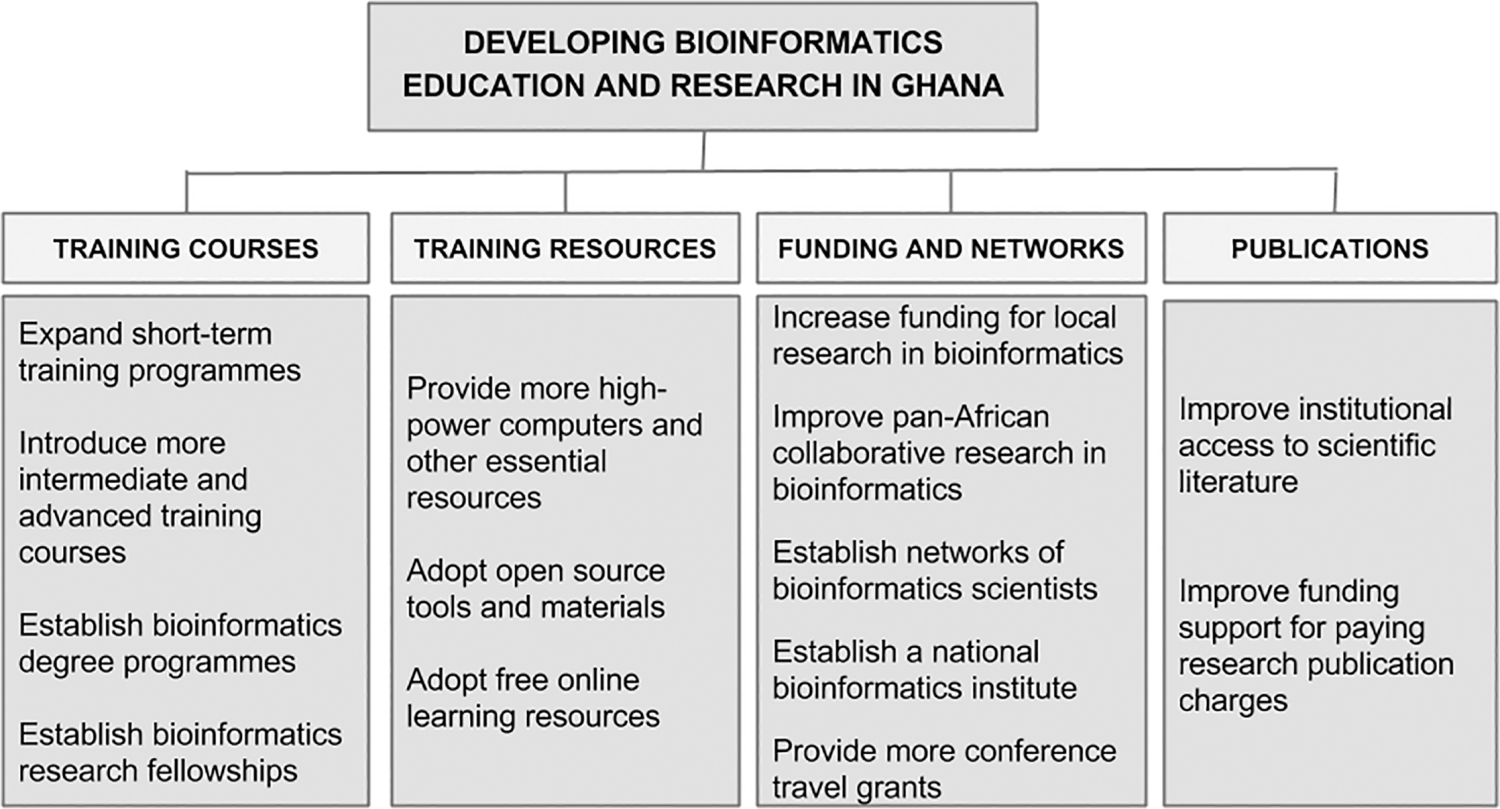
Opportunities for developing bioinformatics in Ghana.

Developing collaborations between Ghanaian scientists could be a useful mechanism to build bioinformatics expertise [13,38]. This approach would present new opportunities, for instance, in collaborative research and student training [1,13,14,38]. Developing collaborative research efforts between scientists in Ghana and their colleagues elsewhere in Africa may also be a sustainable means to boost bioinformatics activities, by pooling together Africa-based resources and expertise to address research questions. This would also help to overcome the lack of intracontinental bioinformatics research collaboration in Ghana; publications recorded in S2 Table were dominated by intercontinental examples. Furthermore, the establishment of a national bioinformatics institute in Ghana, possibly modelled on the well-recognised South African National Bioinformatics Institute (http://www.sanbi.ac.za), would support local research and improve excellence in biology education (Fig 2; [18]). Beyond networks, scientists need to stay current with the global trends in their research areas at all times [70,71]. A key problem affecting science in Africa is the inability of many scientists to attend major international conferences, sometimes due to lack of funding [70]. New discoveries are often discussed at such conferences; missing these events can be costly, particularly in bioinformatics, in which new techniques are frequently introduced. More travel grants and conference scholarships are therefore needed for African peers [70].

### Training opportunities in bioinformatics are limited

Although the approach of offering short courses and workshops is laudable, this model of teaching bioinformatics broadly can hardly produce scientists with expert knowledge in the discipline [72]. For example, participants at introductory bioinformatics courses are usually taught how to compare protein and nucleic acid sequences using alignment tools such as the Basic Local Alignment Search Tool [73]. With these tools, beginners are often content with finding sequence matches, without necessarily understanding the underlying principles [74]. Another issue is that bioinformatics training programmes in Ghana appear to be limited to only a few institutions that have some bioinformatics capacity, leaving behind the majority of institutions. However, to improve the use of bioinformatics may require the expansion of training partnerships to RHEIs that currently lack capacity in the field. Degree programmes and other forms of advanced training (such as research fellowships) will also be essential to provide learners with in-depth skills in bioinformatics [72].

Knowledge in bioinformatics may be essential for the development of many biologists [75]. For this reason, the bioinformatics short courses offered in the country deserve to be continued to provide fundamental knowledge to beginners [1,66,74]. However, these programmes should be supported with more advanced training courses, to help build the computational capacity of biologists (Fig 2; [74]). Furthermore, introducing bioinformatics into degree curricula would help to develop the multidisciplinary skills of students and allow them time to learn about both the use and development of bioinformatics tools [1,33,66]. Interdisciplinary bioinformatics programmes could provide avenues for biologists to complement their biological knowledge with skills in the computational sciences while affording computational science students the opportunity to improve upon their biological knowledge [72,76]. Also, doctoral and postdoctoral research fellowships in bioinformatics should be introduced to help more scientists develop their careers to become independent investigators [77]. S3 Table lists some bioinformatics training programmes and other resources that may be useful in the Ghanaian setting. Also, H3ABioNet has established an African Bioinformatics Education Committee to support bioinformatics curriculum development in Africa [13]. The existing H3ABioNet nodes in Ghana could take advantage of this support system to spearhead the establishment of long-term bioinformatics training programmes.

Ghanaian scientists can also take advantage of the bioinformatics training programmes organised by scientific nonprofit organisations for African scientists. One of the organisations helping to develop bioinformatics in Africa is Teaching and Research in Natural Sciences for Development in Africa (TReND in Africa; http://trendinafrica.org) [68]. An impressive aspect of this organisation’s training programmes is that their volunteers collect surplus equipment in good working condition from Western laboratories and companies to build new laboratories for the courses held at African universities. After the programme, the equipment is donated to the host institution to support local research activities and capacity development [68,78].

### Funding for bioinformatics research is lacking in Ghana

Funding for scientific research remains a key problem in Ghana, especially funding from local agencies. Government funding for research is low; scientific research and development (R&D) receives about 0.3% of the country’s gross domestic product (GDP) [79]. This is less than half the GDP spent in other African countries, such as South Africa, where about 0.87% of GDP is spent on local scientific research [80]. Research funding from the Government of Ghana (GoG) also falls below the African Union’s recommendation that a minimum of 1.0% of national budgets of member states should be spent on local R&D [19]. Only a small fraction of the limited funds (about a tenth of the 0.3% of Ghana’s GDP) goes to support actual research costs because the vast majority (over 90%) is spent on salaries, remunerations, and other operational expenses [79]. Research in the country is therefore highly dependent on international donors [19,79]. This insufficient financial commitment from local stakeholders makes it difficult to ensure the application of modern scientific approaches such as bioinformatics. It is also worth noting that there exists no clear coordination between GoG and the RHEIs (which are the major STI service providers in the country) for setting research priorities and for implementing, administering, and reviewing research funding schemes [79]. There is no clearly defined agency that is responsible for awarding and overseeing competitive research funding in the country, unlike South Africa’s National Research Foundation. As a result, funding directions from GoG to academic institutions often disagree with the research priorities of these institutions and their employee scientists [79], making it challenging for contemporary disciplines like bioinformatics to obtain governmental funding support. Although GoG recently proposed to establish a national research fund, this fund is yet to be operational, and the priority areas remain unknown.

To increase excellence in bioinformatics research will require improvements in funding, especially from local bodies [79]. The GoG’s expenditure on research, particularly in fields that utilise bioinformatics techniques, needs to improve [79]. Other sources of local research funding, such as private companies and internal university funds, should also be explored by scientists [81]. Moreover, it is important for scientists to be more involved in public engagement activities, in order to better communicate the significance of their research to the public and policy makers [81]. This could help to educate the public on the need to invest in scientific research, especially bioinformatics [78].

## Conclusion

Bioinformatics is gradually gaining roots in Ghana. Whilst the capacity for bioinformatics research and training is limited, efforts have been made in the last decade to employ bioinformatics techniques in research targeted at local challenges in biomedical science and agriculture. These developments show that when given the needed support, resource-limited countries like Ghana can contribute to the use of bioinformatics.

It is hoped that the identification of local institutions with expertise in bioinformatics (S1 Table) will help to build partnerships to develop peer support systems among scientists and scientific organisations in Ghana. Furthermore, recognising Ghana’s capacity in bioinformatics can inform science policy directions in order to use the existing capabilities as a foundation and build on this foundation to address other scientific questions. Additionally, identifying bioinformatics-focused research areas should help to build stronger theme-specific alliances, helping to approach local research questions more collectively. Importantly, to ensure the development of the discipline requires that the key challenges of low funding, inadequate training opportunities, and inadequate teaching and research tools are effectively addressed.

## Supporting Information

S1 Table Institutions that conduct bioinformatics-related research in Ghana. (DOCX)

S2 Table Bioinformatics-related peer-reviewed journal articles published by scientists from Ghana (2004–2014). (DOCX)

S3 Table Recommendations to improve bioinformatics in Ghana. (DOCX)
