# Supplementary material for: Bioinformatics in Africa: The Rise of Ghana?
Source: PLoS Comput Biol. 2015 Sep 17;11(9):e1004308. doi: 10.1371/journal.pcbi.1004308 (PMC4574930; doi:10.1371/journal.pcbi.1004308)
Supplement: S1 Table — (DOCX) [file pcbi.1004308.s001.docx]

## S1 Table. Institutions that conduct bioinformatics-related research in Ghana.

This was generated based on research articles published between the periods of 2004 and 2014 and indexed in the PubMed, Web of Science and SCOPUS databases. The search terms “next-generation sequencing Ghana”, “computational biology Ghana”, “bioinformatics Ghana”, “genomic Ghana”, and “*in silico* Ghana” were used to obtain indexed publications from the databases (refer to the main text for article inclusion conditions).

| Institution (website^a^) | Bioinformatics-related research focus^c^ | Bioinformatics-related publications^d^ |
| --- | --- | --- |
| Noguchi Memorial Institute for Medical Research (<http://www.noguchimedres.org>) | Tuberculosis – genotyping and environmental hosts; NTDs; genetic polymorphisms and resistance associated with malaria; genetic risks for glaucoma; characteristics of hepatitis B virus in Ghana; genetic resistance to *Onchocerca volvulus;* identification of a human-bovine reassortment rotavirus strain in Ghana | Dennis et al., 2014; Liu et al., 2013; Osei-Atweneboana et al., 2013; Adu et al., 2012; Soulama et al., 2011; Alam et al., 2011; Gyan et al., 2004; Hilty et al., 2006; Huy et al., 2006; Kaser et al., 2009; Qi et al., 2009; Röltgen et al., 2010 |
| Kintampo Health Research Centre (<http://www.kintampo-hrc.org>) | Modelling malaria infections | Sama et al., 2005 |
| Council for Scientific and Industrial Research (CSIR) – Water Research, Animal Research, Plant Genetic Resources Research, and Crops Research Institutes (<http://www.csir-water.com>; <http://www.csir-ari.org>; http://www.cropsresearch.org) | Genetic resistance to *Onchocerca volvulus;* genomic sequencing of peste des petits ruminant virus; diversity of cowpea accessions in Ghana; mapping maize streak virus distribution across Ghana | Osei-Atweneboana et al., 2013; Dundon et al., 2014; Egbadzor et al., 2014; Oppong et al., 2014 |
| University for Development Studies (<http://www.uds.edu.gh>) | Allelic polymorphisms associated with malaria; pro-inflammatory host responses in adverse environments | Cramer et al., 2004; Kuningas et al., 2009 |
| University of Ghana, and Korle Bu Teaching Hospital^b^ (<http://www.ug.edu.gh>; <http://kbth.gov.gh>) | Genome-wide studies of susceptibilities to prostate cancer; genetic risk factors for glaucoma; genomic sequencing of viruses that cause cassava mosaic disease; transmission of cacao pollen shoot viruses; polymorphisms associated with ahaptoglobinaemia; genetic structure of cocoyam accessions in Ghana; genetic susceptibility to diabetes mellitus; genetic predisposition to hypertension; genome-wide traits associated with obesity; genome-wide traits associated with intraocular pressure; genome-wide investigation of renal function phenotypes; polymorphisms in HIV-1 strains; genomic variants among Ghanaians; genetic diversity of village chickens across Ghana; genomic sequencing of peste des petits ruminant virus; diversity of cowpea accessions in Ghana; mapping maize streak virus distribution across Ghana | Chung et al., 2014; Ali Amin et al., 2014; Liu et al., 2013; Oteng-Frimpong et al., 2012; Muller and Sackey, 2005; Teye et al., 2004; Offei et al., 2004; Rotimi et al., 2004; Williams et al., 2004; Adeyemo et al., 2005; Chen et al., 2005a,b; Rotimi et al., 2006; Chen et al., 2007a,b; Sagoe et al., 2007; Sagoe et al., 2009; Liu et al., 2008; Mita et al., 2009; Yen-Revollo et al., 2009; Osei-Amponsah et al., 2010; Dundon et al., 2014; Egbadzor et al., 2014; Oppong et al., 2014 |
| Biotechnology and Nuclear Agriculture Research Institute, Ghana Atomic Energy Commission (BNARI-GAEC; <http://bnari.gaecgh.org>) | Genetic basis of insecticide resistance in mosquitoes; evolution of *Anopheles gambiae* species | Lynd et al., 2010; Clarkson et al., 2014 |
| Navrongo Health Research Centre (<http://navrongo-hrc.org>) | Genetic polymorphisms associated with severe malaria; epidemiology of Neisseria meningitidis infections in Africa | Auburn et al., 2008; Lamelas et al., 2014 |
| Kwame Nkrumah University of Science and Technology, and Komfo Anokye Teaching Hospital^b^ (<http://www.knust.edu.gh>; <http://www.kathhsp.org>) | Genetic susceptibility to diabetes mellitus; Molecular epidemiology of HIV/AIDS and hepatitits B infections; genetic susceptibility to, and polymorphisms associated with, tuberculosis; genetic protection from tuberculosis; genetic resistance to malaria; diversity of mitochondrial DNA in Ghanaian populations; genetic polymorphisms associated with malaria; genome-wide traits associated with obesity; genome-wide traits associated with intraocular pressure; mother-to-child transmission of hepatitis B infections; genome-wide investigation of renal function phenotypes | Adu et al., 2013; Thye et al., 2012; Thye et al., 2009; Timmann et al., 2012; Fendt et al., 2012; Schuldt et al., 2011; Intemann et al., 2011; Fischetti et al., 2004; Rotimi et al., 2004; Adeyemo et al., 2005; Chen et al., 2005a,b; Rotimi et al., 2006; Candotti et al., 2007; Chen et al., 2007a,b; Timmann et al., 2007; Auburn et al., 2008; Zahn et al., 2008; Intemann et al, 2009; Thye et al., 2010 |
| Ghana Health Service (<http://www.ghanahealthservice.org>) | Genetic diversities associated with tuberculosis; genetic protection from tuberculosis | Hilty et al., 2006; Auburn et al., 2008; Intemann et al, 2009; Röltgen et al., 2010; Thye et al., 2010 |
| Technoserve - Ghana, Accra | Genetic diversity of the shea tree | Fontaine et al., 2004 |
| Kumasi Centre for Collaborative Research in Tropical Medicine (<http://kccr-ghana.org>) | Betacoronaviruses-related viruses in bats; genetic susceptibility to, and protection from tuberculosis; polymorphisms associated with tuberculosis; genetic resistance to malaria; diversity of mitochondrial DNA in Ghanaian populations; genetic resistance to *Onchocerca volvulus* | Intemann et al., 2011; Thye et al., 2012; Timmann et al., 2012; Thye et al., 2009; Fendt et al., 2012; Annan et al, 2013; Timmann et al., 2007; Auburn et al., 2008; Timmann et al., 2008; Intemann et al, 2009; Thye et al., 2010 |
| University of Cape Coast (<http://www.ucc.edu.gh>) | Evolution of *Anopheles gambiae* species | Clarkson et al., 2014 |
| Cocoa Research Institute of Ghana (CRIG) | Diversity of cowpea accessions in Ghana | Egbadzor et al., 2014 |

^a^Institutional websites available as of 10^th^ June, 2015.

^b^Korle Bu and Komfo Anokye hospitals are teaching hospitals affiliated to the medical schools of the University of Ghana (UG) and Kwame Nkrumah University of Science and Technology (KNUST) respectively.

^c^Bioinformatics-related research foci were generated from recent peer-reviewed publications (2004 – 2014) emanating from the various institutions.

^d^For full details of these publications, refer to S2 Table.

Abbreviations: HIV, Human Immunodeficiency Virus; AIDS, Acquired Immunodeficiency Syndrome; DNA, Deoxyribonucleic Acid; NTDs, Neglected Tropical Diseases.
