# Supplementary material for: Bioinformatics in Africa: The Rise of Ghana?
Source: PLoS Comput Biol. 2015 Sep 17;11(9):e1004308. doi: 10.1371/journal.pcbi.1004308 (PMC4574930; doi:10.1371/journal.pcbi.1004308)
Supplement: S2 Table — (DOCX) [file pcbi.1004308.s002.docx]

**S2 Table. Bioinformatics-related peer-reviewed journal articles published by scientists from Ghana (2004 – 2014)^a,b^**

| Year | Article Title | Authors | Citation | PubMed ID | PubMed weblink | Journal weblink |
| --- | --- | --- | --- | --- | --- | --- |
| 2004 | Allelic dimorphism of the erythrocyte binding antigen-175 (eba-175) gene of Plasmodium falciparum and severe malaria: Significant association of the C-segment with fatal outcome in Ghanaian children. | Cramer JP, Mockenhaupt FP, Möhl I, Dittrich S, Dietz E, Otchwemah RN, Ehrhardt S, Bienzle U, Jelinek T | Malar J 3: 11. | 15140262 | <http://www.ncbi.nlm.nih.gov/pmc/articles/PMC420250/> | <http://www.malariajournal.com/content/3/1/11> |
|  | Molecular epidemiology of HIV in Ghana: dominance of CRF02_AG. | Fischetti L, Opare-Sem O, Candotti D, Sarkodie F, Lee H, Allain JP | J Med Virol 73:158–166. | 15122787 | <http://www.ncbi.nlm.nih.gov/pubmed/15122787> | <http://onlinelibrary.wiley.com/doi/10.1002/jmv.20070/abstract> |
|  | Genetic diversity of the shea tree (*Vitellaria paradoxa C.F. Gaertn*), detected by RAPD and chloroplast microsatellite markers. | Fontaine C, Lovett PN, Sanou H, Maley J, Bouvet J-M | Heredity (Edinb) 2004, 93:639–648. | 15354195 | <http://www.ncbi.nlm.nih.gov/pubmed/15354195> | <http://www.nature.com/hdy/journal/v93/n6/full/6800591a.html> |
|  | Allelic polymorphisms in the repeat and promoter regions of the interleukin-4 gene and malaria severity in Ghanaian children. | Gyan BA, Goka B, Cvetkovic JT, Kurtzhals JL, Adabayeri V, Perlmann H, Lefvert A-K, Akanmori BD, Troye-Blomberg M | Clin Exp Immunol 2004, 138:145–150. | 15373917 | <http://www.ncbi.nlm.nih.gov/pubmed/15373917> | <http://onlinelibrary.wiley.com/doi/10.1111/j.1365-2249.2004.02590.x/abstract> |
|  | Genetic structure of seventy cocoyam (*Xanthosoma sagittifolium*, Linn, Schott) accessions in Ghana based on RAPD. | Offei SK, Asante IK, Danquah EY | Hereditas 2004, 140:123–128. | 15061789 | <http://www.ncbi.nlm.nih.gov/pubmed/15061789> | <http://onlinelibrary.wiley.com/doi/10.1111/j.1601-5223.2004.01725.x/abstract> |
|  | A genome-wide search for type 2 diabetes susceptibility genes in West Africans: the Africa America Diabetes Mellitus (AADM) Study. | Rotimi CN, Chen G, Adeyemo AA, Furbert-Harris P, Parish-Gause D, Zhou J, Berg K, Adegoke O, Amoah A, Owusu S, Acheampong J, Agyenim-Boateng K, Eghan BA Jr, Oli J, Okafor G, Ofoegbu E, Osotimehin B, Abbiyesuku F, Johnson T, Rufus T, Fasanmade O, Kittles R, Daniel H, Chen Y, Dunston G, Collins FS, Guass D, Africa America Diabetes Mellitus (AADM) Study | Diabetes 53:838–841. | 14988271 | <http://www.ncbi.nlm.nih.gov/pubmed/14988271> | <http://diabetes.diabetesjournals.org/content/53/3/838.long> |
|  | A novel I247T missense mutation in the haptoglobin 2 beta-chain decreases the expression of the protein and is associated with ahaptoglobinemia. | Teye K, Quaye IKE, Koda Y, Soejima M, Pang H, Tsuneoka M, Amoah AGB, Adjei A, Kimura H | Hum Genet 114:499–502. | 14999562 | <http://www.ncbi.nlm.nih.gov/pubmed/14999562> | <http://link.springer.com/article/10.1007%2Fs00439-004-1098-6> |
|  | Multilocus analysis of hypertension: a hierarchical approach. | Williams SM, Ritchie MD, Phillips JA, Dawson E, Prince M, Dzhura E, Willis A, Semenya A, Summar M, White BC, Addy JH, Kpodonu J, Wong L-J, Felder RA, Jose PA, Moore JH | Hum Hered 57:28–38. | 15133310 | <http://www.ncbi.nlm.nih.gov/pubmed/15133310> | <http://www.karger.com/Article/FullText/77387> |
| 2005 | A genome wide quantitative trait linkage analysis for serum lipids in type 2 diabetes in an African population. | Adeyemo AA, Johnson T, Acheampong J, Oli J, Okafor G, Amoah A, Owusu S, Agyenim-Boateng K, Eghan BA Jr, Abbiyesuku F, Fasanmade O, Rufus T, Doumatey A, Chen G, Zhou J, Chen Y, Furbert-Harris P, Dunston G, Collins F, Rotimi C | Atherosclerosis 181:389–397. | 16039295 | <http://www.ncbi.nlm.nih.gov/pubmed/16039295> | <http://www.atherosclerosis-journal.com/article/S0021-9150(05)00096-1/abstract> |
|  | A genome-wide scan for quantitative trait loci linked to obesity phenotypes among West Africans. | Chen G, Adeyemo AA, Johnson T, Zhou J, Amoah A, Owusu S, Acheampong J, Agyenim-Boateng K, Eghan BA, Oli J, Okafor G, Abbiyesuku F, Dunston GM, Chen Y, Collins F, Rotimi C | Int J Obes (Lond) 29:255–259. | 15611782 | <http://www.ncbi.nlm.nih.gov/pubmed/15611782> | <http://www.nature.com/ijo/journal/v29/n3/full/0802873a.html> |
|  | Calpain-10 gene polymorphisms and type 2 diabetes in West Africans: the Africa America Diabetes Mellitus (AADM) Study. | Chen Y, Kittles R, Zhou J, Chen G, Adeyemo A, Panguluri RK, Chen W, Amoah A, Opoku V, Acheampong J, Agyenim-Boateng K, Eghan BA Jr, Nyantaki A, Oli J, Okafor G, Ofoegbu E, Osotimehin B, Abbiyesuku F, Johnson T, Fasanmade O, Rufus T, Furbert-Harris P, Daniel HI, Berg KA, Collins FS, Dunston GM, Rotimi CN | Ann Epidemiol 15:153–159. | 15652721 | <http://www.ncbi.nlm.nih.gov/pubmed/15652721> | <http://www.annalsofepidemiology.org/article/S1047-2797(04)00091-2/abstract> |
|  | Molecular variability analysis of five new complete cacao swollen shoot virus genomic sequences. | Muller E, Sackey S | Arch Virol 150:53–66. | 15338327 | <http://www.ncbi.nlm.nih.gov/pubmed/15338327> | <http://link.springer.com/article/10.1007%2Fs00705-004-0394-8> |
|  | An immigration-death model to estimate the duration of malaria infection when detectability of the parasite is imperfect. | Sama W, Owusu-Agyei S, Felger I, Vounatsou P, Smith T | Stat Med 24:3269–3288. | 16143990 | <http://www.ncbi.nlm.nih.gov/pubmed/16143990> | <http://onlinelibrary.wiley.com/doi/10.1002/sim.2189/abstract> |
| 2006 | Genetic diversity in Mycobacterium ulcerans isolates from Ghana revealed by a newly identified locus containing a variable number of tandem repeats. | Hilty M, Yeboah-Manu D, Boakye D, Mensah-Quainoo E, Rondini S, Schelling E, Ofori-Adjei D, Portaels F, Zinsstag J, Pluschke G | J Bacteriol 188:1462–1465. | 16452429 | <http://www.ncbi.nlm.nih.gov/pubmed/16452429> | <http://jb.asm.org/content/188/4/1462.long> |
|  | Characteristics of hepatitis B virus in Ghana: full length genome sequences indicate the endemicity of genotype E in West Africa. | Huy TTT, Ishikawa K, Ampofo W, Izumi T, Nakajima A, Ansah J, Tetteh JO, Nii-Trebi N, Aidoo S, Ofori-Adjei D, Sata T, Ushijima H, Abe K | J Med Virol 78:178–184. | 16372296 | <http://www.ncbi.nlm.nih.gov/pubmed/16372296> | <http://onlinelibrary.wiley.com/doi/10.1002/jmv.20525/abstract> |
|  | Genomewide scan and fine mapping of quantitative trait loci for intraocular pressure on 5q and 14q in West Africans. | Rotimi CN, Chen G, Adeyemo AA, Jones LS, Agyenim-Boateng K, Eghan BA Jr, Zhou J, Doumatey A, Lashley K, Huang H, Fasanmade O, Akinsola FB, Ezepue F, Amoah A, Akafo S, Chen Y, Oli J, Johnson T | Invest Ophthalmol Vis Sci 47:3262–3267. | 16877390 | <http://www.ncbi.nlm.nih.gov/pubmed/16877390> | <http://www.iovs.org/content/47/8/3262.long> |
| 2007 | Maternofetal transmission of hepatitis B virus genotype E in Ghana, west Africa. | Candotti D, Danso K, Allain J-P | J Gen Virol 88(Pt 10):2686–2695. | 17872520 | <http://www.ncbi.nlm.nih.gov/pubmed/17872520> | <http://vir.sgmjournals.org/content/88/10/2686.full.pdf+html> |
|  | Genome-wide search for susceptibility genes to type 2 diabetes in West Africans: potential role of C-peptide. | Chen G, Adeyemo A, Zhou J, Chen Y, Huang H, Doumatey A, Lashley K, Agyenim-Boateng K, Eghan BA Jr, Acheampong J, Fasanmade O, Johnson T, Okafor G, Oli J, Amoah A, Rotimi C | Diabetes Res Clin Pract 78:e1–6. | 17548123 | <http://www.ncbi.nlm.nih.gov/pubmed/17548123> | <http://www.sciencedirect.com/science/article/pii/S0168822707002999> |
|  | A genome-wide search for linkage to renal function phenotypes in West Africans with type 2 diabetes. | Chen G, Adeyemo AA, Zhou J, Chen Y, Doumatey A, Lashley K, Huang H, Amoah A, Agyenim-Boateng K, Eghan BA Jr, Okafor G, Acheampong J, Oli J, Fasanmade O, Johnson T, Rotimi C | Am J Kidney Dis 49:394–400. | 17336700 | <http://www.ncbi.nlm.nih.gov/pubmed/17336700> | <http://www.ajkd.org/article/S0272-6386(06)01912-3/abstract> |
|  | Variability of the human immunodeficiency virus type 1 polymerase gene from treatment naïve patients in Accra, Ghana. | Sagoe KWC, Dwidar M, Lartey M, Boamah I, Agyei AA, Hayford AA, Mingle JAA, Arens MQ | J Clin Virol 40:163–167. | 17827059 | <http://www.ncbi.nlm.nih.gov/pubmed/17827059> | [http://www.sciencedirect.com/science/article/pii/S1386653207002624#](http://www.sciencedirect.com/science/article/pii/S1386653207002624) |
|  | Genome-wide linkage analysis of malaria infection intensity and mild disease. | Timmann C, Evans JA, König IR, Kleensang A, Rüschendorf F, Lenzen J, Sievertsen J, Becker C, Enuameh Y, Kwakye KO, Opoku E, Browne ENL, Ziegler A, Nürnberg P, Horstmann RD | PLoS Genet 3:e48. | 17381244 | <http://www.ncbi.nlm.nih.gov/pubmed/17381244> | <http://www.plosgenetics.org/article/info%3Adoi%2F10.1371%2Fjournal.pgen.0030048> |
| 2008 | Association of the GNAS locus with severe malaria. | Auburn S, Diakite M, Fry AE, Ghansah A, Campino S, Richardson A, Jallow M, Sisay-Joof F, Pinder M, Griffiths MJ, Peshu N, Williams TN, Marsh K, Molyneux ME, Taylor TE, Koram KA, Oduro AR, Rogers WO, Rockett KA, Haldar K, Kwiatkowski DP | Hum Genet 124:499–506. | 18951142 | <http://www.ncbi.nlm.nih.gov/pubmed/18951142> | <http://link.springer.com/article/10.1007%2Fs00439-008-0575-8> |
|  | ALOX5 variants associated with susceptibility to human pulmonary tuberculosis. | Herb F, Thye T, Niemann S, Browne ENL, Chinbuah MA, Gyapong J, Osei I, Owusu-Dabo E, Werz O, Rüsch-Gerdes S, Horstmann RD, Meyer CG | Hum Mol Genet 17:1052–1060. | 18174194 | <http://www.ncbi.nlm.nih.gov/pubmed/18174194> | <http://hmg.oxfordjournals.org/content/17/7/1052.long> |
|  | Optineurin coding variants in Ghanaian patients with primary open-angle glaucoma. | Liu Y, Akafo S, Santiago-Turla C, Cohen CS, Larocque-Abramson KR, Qin X, Herndon LW, Challa P, Schmidt S, Hauser MA, Allingham RR | Mol Vis 14:2367–2372. | 19096531 | <http://www.ncbi.nlm.nih.gov/pubmed/19096531> | <http://www.molvis.org/molvis/v14/a273/> |
|  | Human genetic resistance to Onchocerca volvulus: Evidence for linkage to chromosome 2p from an autosome-wide scan. | Timmann C, Van Der Kamp E, Kleensang A, König IR, Thye T, Büttner DW, Hamelmann C, Marfo Y, Vens M, Brattig N, Ziegler A, Horstmann RD | J Infect Dis 198:427–433. | 18558870 | <http://www.ncbi.nlm.nih.gov/pubmed/18558870> | <http://jid.oxfordjournals.org/content/198/3/427.long> |
|  | Molecular characterization of occult hepatitis B virus in genotype E-infected subjects. | Zahn A, Li C, Danso K, Candotti D, Owusu-Ofori S, Temple J, Allain J-P | J Gen Virol 89(Pt 2):409–418. | 18198371 | <http://www.ncbi.nlm.nih.gov/pubmed/18198371> | <http://vir.sgmjournals.org/content/89/2/409.long> |
| 2009 | Autophagy gene variant IRGM -261T contributes to protection from tuberculosis caused by Mycobacterium tuberculosis but not by M. africanum strains. | Intemann CD, Thye T, Niemann S, Browne ENL, Amanua Chinbuah M, Enimil A, Gyapong J, Osei I, Owusu-Dabo E, Helm S, Rüsch-Gerdes S, Horstmann RD, Meyer CG | PLoS Pathog 5:e1000577. | 19750224 | <http://www.ncbi.nlm.nih.gov/pubmed/19750224> | <http://www.plospathogens.org/article/info%3Adoi%2F10.1371%2Fjournal.ppat.1000577> |
|  | Lack of insertional-deletional polymorphism in a collection of Mycobacterium ulcerans isolates from Ghanaian Buruli ulcer patients. | Käser M, Gutmann O, Hauser J, Stinear T, Cole S, Yeboah-Manu D, Dernick G, Certa U, Pluschke G | J Clin Microbiol 47:3640–3646. | 19726605 | <http://www.ncbi.nlm.nih.gov/pubmed/19726605> | <http://jcm.asm.org/content/47/11/3640.long> |
|  | Selection for genetic variation inducing pro-inflammatory responses under adverse environmental conditions in a Ghanaian population. | Kuningas M, May L, Tamm R, van Bodegom D, van den Biggelaar AHJ, Meij JJ, Frölich M, Ziem JB, Suchiman HED, Metspalu A, Slagboom PE, Westendorp RGJ | PLoS ONE 4:e7795. | 19907653 | <http://www.ncbi.nlm.nih.gov/pubmed/19907653> | <http://www.plosone.org/article/info%3Adoi%2F10.1371%2Fjournal.pone.0007795> |
|  | Indigenous evolution of Plasmodium falciparum pyrimethamine resistance multiple times in Africa. | Mita T, Tanabe K, Takahashi N, Culleton R, Ndounga M, Dzodzomenyo M, Akhwale WS, Kaneko A, Kobayakawa T | J Antimicrob Chemother 63:252–255. | 19036751 | <http://www.ncbi.nlm.nih.gov/pubmed/19036751> | <http://jac.oxfordjournals.org/content/63/2/252.long> |
|  | Genomic diversity and evolution of Mycobacterium ulcerans revealed by next-generation sequencing. | Qi W, Käser M, Röltgen K, Yeboah-Manu D, Pluschke G | PLoS Pathog 5:e1000580. | 19806175 | <http://www.ncbi.nlm.nih.gov/pubmed/19806175> | <http://www.plospathogens.org/article/info%3Adoi%2F10.1371%2Fjournal.ppat.1000580> |
|  | HIV-1 CRF 02 AG polymerase genes in Southern Ghana are mosaics of different 02 AG strains and the protease gene cannot infer subtypes. | Sagoe KW, Dwidar M, Adiku TK, Arens MQ | Virol J 6:27. | 19245688 | <http://www.ncbi.nlm.nih.gov/pubmed/19245688> | <http://www.virologyj.com/content/6/1/27> |
|  | MCP-1 promoter variant -362C associated with protection from pulmonary tuberculosis in Ghana, West Africa. | Thye T, Nejentsev S, Intemann CD, Browne EN, Chinbuah MA, Gyapong J, Osei I, Owusu-Dabo E, Zeitels LR, Herb F, Horstmann RD, Meyer CG | Hum Mol Genet 18:381–388. | 18940815 | <http://www.ncbi.nlm.nih.gov/pubmed/18940815> | <http://hmg.oxfordjournals.org/content/18/2/381.long> |
|  | Influence of ethnicity on pharmacogenetic variation in the Ghanaian population. | Yen-Revollo JL, Van Booven DJ, Peters EJ, Hoskins JM, Engen RM, Kannall HD, Ofori-Adjei D, McLeod HL, Marsh S | Pharmacogenomics J 9:373–379. | 19546880 | <http://www.ncbi.nlm.nih.gov/pubmed/19546880> | <http://www.nature.com/tpj/journal/v9/n6/full/tpj200928a.html> |
| 2010 | Field, genetic, and modeling approaches show strong positive selection acting upon an insecticide resistance mutation in Anopheles gambiae s.s. | Lynd A, Weetman D, Barbosa S, Egyir Yawson A, Mitchell S, Pinto J, Hastings I, Donnelly MJ | Mol Biol Evol 27:1117–1125. | 20056691 | <http://www.ncbi.nlm.nih.gov/pubmed/20056691> | <http://mbe.oxfordjournals.org/content/27/5/1117.long> |
|  | Genetic diversity of Forest and Savannah chicken populations of Ghana as estimated by microsatellite markers. | Osei-Amponsah R, Kayang BB, Naazie A, Osei YD, Youssao IAK, Yapi-Gnaore VC, Tixier-Boichard M, Rognon X | Anim Sci J 81:297–303. | 20597885 | <http://www.ncbi.nlm.nih.gov/pubmed/20597885> | <http://onlinelibrary.wiley.com/doi/10.1111/j.1740-0929.2010.00749.x/abstract> |
|  | Single nucleotide polymorphism typing of Mycobacterium ulcerans reveals focal transmission of buruli ulcer in a highly endemic region of Ghana. | Röltgen K, Qi W, Ruf M-T, Mensah-Quainoo E, Pidot SJ, Seemann T, Stinear TP, Käser M, Yeboah-Manu D, Pluschke G | PLoS Negl Trop Dis 4:e751. | 20652033 | <http://www.ncbi.nlm.nih.gov/pubmed/20652033> | <http://www.plosntds.org/article/info%3Adoi%2F10.1371%2Fjournal.pntd.0000751> |
|  | Genome-wide association analyses identifies a susceptibility locus for tuberculosis on chromosome 18q11.2. | Thye T, Vannberg FO, Wong SH, Owusu-Dabo E, Osei I, Gyapong J, Sirugo G, Sisay-Joof F, Enimil A, Chinbuah MA, Floyd S, Warndorff DK, Sichali L, Malema S, Crampin AC, Ngwira B, Teo YY, Small K, Rockett K, Kwiatkowski D, Fine PE, Hill PC, Newport M, Lienhardt C, Adegbola RA, Corrah T, Ziegler A, African TB Genetics Consortium, Wellcome Trust Case Control Consortium, Morris AP, et al. | Nat Genet 42:739–741. | 20694014 | <http://www.ncbi.nlm.nih.gov/pubmed/20694014> | <http://www.nature.com/ng/journal/v42/n9/full/ng.639.html> |
| 2011 | Selective sweeps and genetic lineages of Plasmodium falciparum drug -resistant alleles in Ghana. | Alam MT, de Souza DK, Vinayak S, Griffing SM, Poe AC, Duah NO, Ghansah A, Asamoa K, Slutsker L, Wilson MD, Barnwell JW, Udhayakumar V, Koram KA | J Infect Dis 203:220–227. | 21288822 | <http://www.ncbi.nlm.nih.gov/pubmed/21288822> | <http://jid.oxfordjournals.org/content/203/2/220.long> |
|  | MCP1 haplotypes associated with protection from pulmonary tuberculosis. | Intemann CD, Thye T, Förster B, Owusu-Dabo E, Gyapong J, Horstmann RD, Meyer CG | BMC Genet 12:34. | 21504590 | <http://www.ncbi.nlm.nih.gov/pubmed/21504590> | <http://www.biomedcentral.com/1471-2156/12/34> |
|  | A -436C>A polymorphism in the human FAS gene promoter associated with severe childhood malaria. | Schuldt K, Kretz CC, Timmann C, Sievertsen J, Ehmen C, Esser C, Loag W, Ansong D, Dering C, Evans J, Ziegler A, May J, Krammer PH, Agbenyega T, Horstmann RD | PLoS Genet 7:e1002066. | 21625619 | <http://www.ncbi.nlm.nih.gov/pubmed/21625619> | <http://www.plosgenetics.org/article/info%3Adoi%2F10.1371%2Fjournal.pgen.1002066> |
|  | Genetic diversity of polymorphic vaccine candidate antigens (apical membrane antigen-1, merozoite surface protein-3, and erythrocyte binding antigen-175) in Plasmodium falciparum isolates from western and central Africa. | Soulama I, Bigoga JD, Ndiaye M, Bougouma EC, Quagraine J, Casimiro PN, Stedman TT, Sirima SB | Am J Trop Med Hyg 84:276–284. | 21292899 | <http://www.ncbi.nlm.nih.gov/pubmed/21292899> | <http://www.ajtmh.org/content/84/2/276.long> |
| 2012 | Fc γ receptor IIIB (FcγRIIIB) polymorphisms are associated with clinical malaria in Ghanaian children. | Adu B, Dodoo D, Adukpo S, Hedley PL, Arthur FKN, Gerds TA, Larsen SO, Christiansen M, Theisen M | PLoS ONE 7:e46197. | 23049979 | <http://www.ncbi.nlm.nih.gov/pubmed/23049979> | <http://www.plosone.org/article/info%3Adoi%2F10.1371%2Fjournal.pone.0046197> |
|  | MtDNA diversity of Ghana: a forensic and phylogeographic view. | Fendt L, Röck A, Zimmermann B, Bodner M, Thye T, Tschentscher F, Owusu-Dabo E, Göbel TMK, Schneider PM, Parson W | Forensic Sci Int Genet 6:244–249. | 21723214 | <http://www.ncbi.nlm.nih.gov/pubmed/21723214> | <http://www.ncbi.nlm.nih.gov/pmc/articles/PMC3314991/> |
|  | Identification and validation of a gene causing cross-resistance between insecticide classes in Anopheles gambiae from Ghana. | Mitchell SN, Stevenson BJ, Müller P, Wilding CS, Egyir-Yawson A, Field SG, Hemingway J, Paine MJI, Ranson H, Donnelly MJ | Proc Natl Acad Sci USA 109:6147–6152. | 22460795 | <http://www.ncbi.nlm.nih.gov/pubmed/22460795> | <http://www.pnas.org/content/109/16/6147.long> |
|  | Genotypic analysis of β-tubulin in Onchocerca volvulus from communities and individuals showing poor parasitological response to ivermectin treatment. | Osei-Atweneboana MY, Boakye DA, Awadzi K, Gyapong JO, Prichard RK | Int J Parasitol Drugs Drug Resist 2:20–28. | 24533268 | <http://www.ncbi.nlm.nih.gov/pubmed/24533268> | <http://www.sciencedirect.com/science/article/pii/S2211320712000061> |
|  | Complete genome sequencing of two causative viruses of cassava mosaic disease in Ghana. | Oteng-Frimpong R, Levy Y, Torkpo SK, Danquah EY, Offei SK, Gafni Y | Acta Virol 56:305–314. | 23237086 | <http://www.ncbi.nlm.nih.gov/pubmed/23237086> | <http://www.elis.sk/index.php?page=shop.product_details&flypage=flypage.tpl&product_id=3217&category_id=98&option=com_virtuemart&vmcchk=1&Itemid=1> |
|  | Large-scale survey for novel genotypes of Plasmodium falciparum chloroquine-resistance gene pfcrt. | Takahashi N, Tanabe K, Tsukahara T, Dzodzomenyo M, Dysoley L, Khamlome B, Sattabongkot J, Nakamura M, Sakurai M, Kobayashi J, Kaneko A, Endo H, Hombhanje F, Tsuboi T, Mita T | Malar J 11:92. | 22453078 | <http://www.ncbi.nlm.nih.gov/pubmed/22453078> | <http://www.ncbi.nlm.nih.gov/pubmed/22453078> |
|  | Common variants at 11p13 are associated with susceptibility to tuberculosis. | Thye T, Owusu-Dabo E, Vannberg FO, van Crevel R, Curtis J, Sahiratmadja E, Balabanova Y, Ehmen C, Muntau B, Ruge G, Sievertsen J, Gyapong J, Nikolayevskyy V, Hill PC, Sirugo G, Drobniewski F, van de Vosse E, Newport M, Alisjahbana B, Nejentsev S, Ottenhoff THM, Hill AVS, Horstmann RD, Meyer CG | Nat Genet 44:257–259. | 22306650 | <http://www.ncbi.nlm.nih.gov/pubmed/22306650> | <http://www.nature.com/ng/journal/v44/n3/full/ng.1080.html> |
|  | Genome-wide association study indicates two novel resistance loci for severe malaria. | Timmann C, Thye T, Vens M, Evans J, May J, Ehmen C, Sievertsen J, Muntau B, Ruge G, Loag W, Ansong D, Antwi S, Asafo-Adjei E, Nguah SB, Kwakye KO, Akoto AOY, Sylverken J, Brendel M, Schuldt K, Loley C, Franke A, Meyer CG, Agbenyega T, Ziegler A, Horstmann RD | Nature 489:443–446. | 22895189 | <http://www.ncbi.nlm.nih.gov/pubmed/22895189> | <http://www.nature.com/nature/journal/v489/n7416/full/nature11334.html> |
| 2013 | Human betacoronavirus 2c EMC/2012-related viruses in bats, Ghana and Europe. | Annan A, Baldwin HJ, Corman VM, Klose SM, Owusu M, Nkrumah EE, Badu EK, Anti P, Agbenyega O, Meyer B, Oppong S, Sarkodie YA, Kalko EKV, Lina PHC, Godlevska EV, Reusken C, Seebens A, Gloza-Rausch F, Vallo P, Tschapka M, Drosten C, Drexler JF | Emerging Infect Dis 19:456–459. | 23622767 | <http://www.ncbi.nlm.nih.gov/pubmed/23622767> | <http://wwwnc.cdc.gov/eid/article/19/3/12-1503_article> |
| 2014 | Evaluation of Customised Lineage-Specific Sets of MIRU-VNTR Loci for Genotyping Mycobacterium tuberculosis Complex Isolates in Ghana. | Asante-Poku A, Nyaho MS, Borrell S, Comas I, Gagneux S, Yeboah-Manu D | PLoS ONE 9:e92675. | 24667333 | <http://www.ncbi.nlm.nih.gov/pubmed/24667333> | <http://www.plosone.org/article/info%3Adoi%2F10.1371%2Fjournal.pone.0092675> |
|  | A genome-wide association study of prostate cancer in West African men. | Cook MB, Wang Oteng-Frimpong Oteng-Frimpong Z, Yeboah ED, Tettey Y, Biritwum RB, Adjei AA, Tay E, Truelove A, Niwa S, Chung CC, Chokkalingam AP, Chu LW, Yeager M, Hutchinson A, Yu K, Rand KA, Haiman CA, Hoover RN, Hsing AW, Chanock SJ, African Ancestry Prostate Cancer GWAS Consortium | Hum Genet 133:509–521. | 24185611 | <http://www.ncbi.nlm.nih.gov/pubmed/24185611> | <http://link.springer.com/article/10.1007%2Fs00439-013-1387-z> |
|  | CYP6 P450 enzymes and ACE-1 duplication produce extreme and multiple insecticide resistance in the malaria mosquito Anopheles gambiae. | Edi CV, Djogbénou L, Jenkins AM, Regna K, Muskavitch MAT, Poupardin R, Jones CM, Essandoh J, Kétoh GK, Paine MJI, Koudou BG, Donnelly MJ, Ranson H, Weetman D | PLoS Genet 10:e1004236. | 24651294 | <http://www.ncbi.nlm.nih.gov/pubmed/24651294> | <http://www.plosgenetics.org/article/info%3Adoi%2F10.1371%2Fjournal.pgen.1004236> |
|  | A meta-analysis of 87,040 individuals identifies 23 new susceptibility loci for prostate cancer. | Al Olama AA, Kote-Jarai Z, Berndt SI, Conti DV, Schumacher F, Han Y, Benlloch S, Hazelett DJ, Wang Z, Saunders E, Leongamornlert D, Lindstrom S, Jugurnauth-Little S, Dadaev T, Tymrakiewicz M, Stram DO, Rand K, Wan P, Stram A, Sheng X, Pooler LC, Park K, Xia L, Tyrer J, Kolonel LN, Le Marchand L, Hoover RN, Machiela MJ, Yeager M, Burdette L, et al. | Nat Genet 46:1103–1109. | 25217961 | <http://www.ncbi.nlm.nih.gov/pubmed/25217961> | <http://www.nature.com/ng/journal/v46/n10/full/ng.3094.html#affil-auth> |
|  | Identification of novel Ghanaian G8P[6] human-bovine reassortant rotavirus strain by next generation sequencing. | Dennis FE, Fujii Y, Haga K, Damanka S, Lartey B, Agbemabiese CA, Ohta N, Armah GE, Katayama K | PLoS ONE 9:e100699. | 24971993 | <http://www.ncbi.nlm.nih.gov/pubmed/24971993> | <http://journals.plos.org/plosone/article?id=10.1371/journal.pone.0100699> |
|  | A comprehensive resequence-analysis of 250 kb region of 8q24.21 in men of African ancestry. | Chung CC, Hsing AW, Edward Yeboah null, Biritwum R, Tettey Y, Adjei A, Cook MB, De Marzo A, Netto G, Tay E, Boland JF, Yeager M, Chanock SJ | Prostate 74:579–589. | 24783269 | <http://www.ncbi.nlm.nih.gov/pubmed/24783269> | <http://onlinelibrary.wiley.com/doi/10.1002/pros.22726/abstract> |
|  | Adaptive introgression between Anopheles sibling species eliminates a major genomic island but not reproductive isolation. | Clarkson CS, Weetman D, Essandoh J, Yawson AE, Maslen G, Manske M, Field SG, Webster M, Antão T, MacInnis B, Kwiatkowski D, Donnelly MJ | Nat Commun 5:4248. | 24963649 | <http://www.ncbi.nlm.nih.gov/pubmed/24963649> | <http://www.nature.com/ncomms/2014/140625/ncomms5248/full/ncomms5248.html> |
|  | Full genome sequence of a peste des petits ruminants virus (PPRV) from Ghana. | Dundon WG, Adombi C, Waqas A, Otsyina HR, Arthur CT, Silber R, Loitsch A, Diallo A: | Virus Genes 49:497–501. | 25150987 | <http://www.ncbi.nlm.nih.gov/pubmed/25150987> | <http://link.springer.com/article/10.1007%2Fs11262-014-1109-1> |
|  | Diversity in 113 cowpea [Vigna unguiculata (L) Walp] accessions assessed with 458 SNP markers. | Egbadzor KF, Ofori K, Yeboah M, Aboagye LM, Opoku-Agyeman MO, Danquah EY, Offei SK | Springerplus 3:541. | 25332852 | <http://www.ncbi.nlm.nih.gov/pubmed/25332852> | <http://www.springerplus.com/content/3/1/541#sec2> |
|  | Emergence of a new epidemic Neisseria meningitidis serogroup A Clone in the African meningitis belt: high-resolution picture of genomic changes that mediate immune evasion. | Lamelas A, Harris SR, Röltgen K, Dangy J-P, Hauser J, Kingsley RA, Connor TR, Sie A, Hodgson A, Dougan G, Parkhill J, Bentley SD, Pluschke G | MBio 5:e01974–01914. | 25336458 | <http://www.ncbi.nlm.nih.gov/pubmed/25336458> | <http://mbio.asm.org/content/5/5/e01974-14.full> |
|  | Mapping the distribution of maize streak virus genotypes across the forest and transition zones of Ghana. | Oppong A, Offei SK, Ofori K, Adu-Dapaah H, Lamptey JNL, Kurenbach B, Walters M, Shepherd DN, Martin DP, Varsani A | Arch Virol DOI 10.1007/s00705-014-2260-7 | 25344899 | <http://www.ncbi.nlm.nih.gov/pubmed/25344899> | <http://link.springer.com/article/10.1007%2Fs00705-014-2260-7> |
|  | Imputation and subset-based association analysis across different cancer types identifies multiple independent risk loci in the TERT-CLPTM1L region on chromosome 5p15.33. | Wang Z, Zhu B, Zhang M, Parikh H, Jia J, Chung CC, Sampson JN, Hoskins JW, Hutchinson A, Burdette L, Ibrahim A, Hautman C, Raj PS, Abnet CC, Adjei AA, Ahlbom A, Albanes D, Allen NE, Ambrosone CB, Aldrich M, Amiano P, Amos C, Andersson U, Andriole G, Andrulis IL, Arici C, Arslan AA, Austin MA, Baris D, Barkauskas DA, et al. | Hum Mol Genet 23:6616–6633. | 25027329 | <http://www.ncbi.nlm.nih.gov/pubmed/25027329> | <http://hmg.oxfordjournals.org/content/23/24/6616> |

^a^The definition of “bioinformatics techniques” in this article followed that of: Luscombe NM, Greenbaum D, Gerstein M: What is bioinformatics? A proposed definition and overview of the field. *Methods Inf Med* 2001, 40:346–358.

^b^Refer to the main text for article inclusion criteria.
