# Supplementary material for: Bioinformatics in Africa: The Rise of Ghana?
Source: PLoS Comput Biol. 2015 Sep 17;11(9):e1004308. doi: 10.1371/journal.pcbi.1004308 (PMC4574930; doi:10.1371/journal.pcbi.1004308)
Supplement: S3 Table — (DOCX) [file pcbi.1004308.s003.docx]

## Table S3. Recommendations to improve bioinformatics in Ghana.

| **Challenge** | **Recommendation** | **Rationale** | **Sample Resources** | | |
| --- | --- | --- | --- | --- | --- |
|  |  |  | **Name (Focus)** | **Website*** | |
| Lack of training opportunities | Extend coverage of introductory courses | To increase basic knowledge in bioinformatics among scientists in Ghana | Introduction to bioinformatics: (introduces basic bioinformatics concepts: from fundamental algorithms to practical tools) | <http://mendel.informatics.indiana.edu/~yye/lab/teaching/fall2013-I519.php> | |
|  |  |  | Bioinformatics and proteomics (hands-on approach to bioinformatics and proteomics) | <http://ocw.mit.edu/courses/electrical-engineering-and-computer-science/6-092-bioinformatics-and-proteomics-january-iap-2005/index.htm> | |
|  |  |  | Introduction to bioinformatics (computational data mining approaches) | <http://ocw.metu.edu.tr/course/view.php?id=37> | |
|  |  |  | Quantitative biology workshop (a workshop-style introduction to biological research tools) | <https://www.edx.org/course/mitx/mitx-7-qbwx-quantitative-biology-1714#.U45b0Bb9O18> | |
|  |  |  | Online lectures on bioinformatics (introduction to basic tools) | <http://lectures.molgen.mpg.de/online_lectures.html> | |
|  | Introduce advanced training courses | To improve specialist knowledge and application of bioinformatics | Data analysis for genomics (harnessing genomics data to answer biological questions) | <https://www.edx.org/course/harvardx/harvardx-ph525x-data-analysis-genomics-1401#.U45b4xb9O18> | |
|  |  |  | Introduction to systems biology (an introduction to contemporary Systems Biology) | <https://www.coursera.org/course/sysbio> | |
|  |  |  | Statistical learning theory and applications (supervised learning from the perspective of modern statistical learning theory) | <http://ocw.mit.edu/courses/brain-and-cognitive-sciences/9-520-statistical-learning-theory-and-applications-spring-2006/index.htm> | |
|  | Bioinformatics degree programmes | To produce local bioinformatics experts | Examples of undergraduate courses in bioinformatics | <http://www.ncl.ac.uk/undergraduate/degrees/i520/courseoverview/>; <http://www.bioinformatics.ucla.edu/undergraduate-courses/> | |
|  |  |  | Examples of masters programmes in bioinformatics | | <http://octette.cs.man.ac.uk/bioinformatics/qualifications/index.html>; <http://advanced.jhu.edu/academics/graduate-degree-programs/bioinformatics/>; <http://mscb.cryst.bbk.ac.uk/modules.html>; <http://www3.imperial.ac.uk/lifesciences/postgraduate/courselist/bioinformatics>; <http://www2.le.ac.uk/departments/biochemistry/postgraduate-study/bioinformatics/msc-bioinformatics-course-details>; <http://www.bu.edu/bioinformatics/admissions/programs/md-track/>; <http://www.brandeis.edu/gps/courses/programs/listings/bioinformatics.html> |
|  |  |  | Examples of PhD programmes in bioinformatics | <http://www.manchester.ac.uk/study/postgraduate-research/programmes/list/05158/bioinformatics-phd/programme-details/>; <http://www.embl.de/training/eipp/index.html>; <http://www.bbk.ac.uk/study/2014/phd/programmes/RMPBIOLG/> | |
|  | Bioinformatics (postdoctoral) research fellowships | To ensure the supply of highly qualified professionals in bioinformatics | Tips for postdoctoral fellowships | <http://sciencecareers.sciencemag.org/career_magazine/previous_issues/articles/2012_08_24/science.opms.r1200121>; <http://biomedicalprograms.georgetown.edu/career-blog/getting-a-postdoc/> | |
|  | Adoption of free, open source learning resources | To allow students to take courses from anywhere, at any time | Examples of free, open source resources | <http://openeuroscience.wordpress.com/>; <http://nba.uth.tmc.edu/snnap/>; <http://www.sanger.ac.uk/resources/software/artemis/>; | |
| Lack of research funding | Increase research funding, especially from local bodies | To support research through funding, human resource development and provision of essential facilities | National Research Foundation, South Africa (an independent government agency mandated to promote and support scientific research through funding) | <http://www.nrf.ac.za> | |
| Lack of bioinformatics networks and institutes | Establish local bioinformatics networks and institutes | To help build capacity in collaborative research and student training. | South African National Bioinformatics Institute; International Society for Computational Biology; African Society of Human Genetics; African Society for Bioinformatics and Computational Biology | <http://www.sanbi.ac.za>; <http://www.iscb.org>; <http://www.afshg.org>; <http://www.asbcb.org> | |

*Accessed 10^th^ June 2015
